# Seroprevalence of Human Herpesvirus 8 and Hepatitis C Virus among Drug Users in Shanghai, China

**DOI:** 10.3390/v6062519

**Published:** 2014-06-23

**Authors:** Tiejun Zhang, Ying Liu, Yuyan Zhang, Jun Wang, Veenu Minhas, Charles Wood, Na He

**Affiliations:** 1 Department of Epidemiology, School of Public Health, Fudan University, and The Key Laboratory of Public Health Safety of Ministry of Education, Shanghai 200032, China; E-Mails: tjzhang@shmu.edu.cn (T.Z.); hailingilei@126.com (Y.L.); 13211020065@fudan.edu.cn (J.W.); 2 Putuo District Center for Disease Control and Prevention, Shanghai 200032, China; E-Mail: zhangyy1@shpt.gov.cn; 3 Nebraska Center for Virology, School of Biological Sciences, University of Nebraska-Lincoln, Lincoln, NE 68583, USA; E-Mails: vminhas2@unl.edu (V.M.); cwood@unl.edu (C.W.)

**Keywords:** human herpesvirus 8, hepatitis C virus, drug user, prevalence, China

## Abstract

To elucidate and compare the seroprevalence of human herpesvirus 8 (HHV8) and hepatitis C virus (HCV) among Chinese drug users, a cross-sectional study of 441 participants, was conducted in Shanghai, China, from 2012 through 2013. Seventy-seven (17.5%) participants were found to be positive for HHV8 antibodies, while 271 (61.5%) participants were positive for HCV. No significant association between HHV8 seropositivity and drug use characteristics, sexual behaviors, HCV, or syphilis was observed. In contrast, a statistically significant association between HCV seropositivity and injected drug history (OR, 2.18, 95% CI 1.41–3.37) was detected, whereas no statistically significant association between HCV seropositivity and syphilis infection (OR, 7.56, 95% CI 0.94–60.57) were observed. Pairwise comparisons showed no significant differences between latent and lytic antibodies regarding HCV and HHV8 serostatus. The study demonstrated a moderate but elevated prevalence of HHV8 infection among drug users. The discordance between HHV8 and HCV infections suggests that blood borne transmission of HHV8 might not be the predominant mode of transmission in this population, which is in contrast to HCV.

## 1. Introduction

Human herpesvirus 8 (HHV8), also known as Kaposi’s Sarcoma associated herpesvirus (KSHV), was discovered in 1994 and is now known to be the etiologic agent for Kaposi’s sarcoma (KS), primary effusion lymphoma (PEL), and multicentric Castleman’s disease [1,2,3,4]. Since its initial discovery, the HHV8 epidemiology has been widely studied globally, showing significant differences in distribution among various geographical areas and various populations analyzed. A number of epidemiological studies have indicated that HHV8 seroprevalence vary considerably among countries and risk groups, but the routes of transmission have yet to be clearly defined [5]. Nonsexual and vertical transmission routes are believed to be of importance in endemic areas in a number of African countries, and a number of studies have demonstrated that saliva contact may be the major mode of transmission [6,7]. Sexual transmission has been shown to occur frequently among homosexual men in non-endemic areas, such as United States and Western Europe [8]. However, evidence of transmission via blood contact remains controversial [9,10,11]. The possibility that HHV8 can be transmitted by blood contact raises important public health concerns. Of particular concern are drug users, since the possibility of blood transmission may facilitate HHV8 transmission, and increase the prevalence of infection among this population. Evidence of blood borne transmission of HHV8 among injection-drug users has been suggested previously, but the data generated are from different population and could not exclude the possibility that the virus is transmitted via other modes of infection, such as via sexual contact and/or general behaviors accompanying the use of drugs rather than the drug use itself [12,13,14].

Drug users have been well documented to be at high risk of blood borne infections (e.g., hepatitis C virus) in China [15,16], but seroprevalence of HHV8 among drug users remained poorly documented, especially in China where there is a large population of intravenous drug users, and they are known to be at risk for Human immunodeficiency virus (HIV) and other associating co-infections. Prior studies among drug users have associated injection drug use practices with higher prevalence of HHV8 infection but with inconsistent results regarding the possibility of HHV8 transmission through drug use. Whether this route of transmission can efficiently occur among this population remains controversial [13,14,17,18]. Moreover, this route of transmission has not yet been evaluated in China, therefore, we recruited a group of drug users which has provided us with the unique opportunity to assess the possibility of HHV8 blood transmission through injection drug use practices. We hypothesized that if HHV8 can be transmitted through blood contact like hepatitis C virus (HCV), the seroprevalence of HHV8 should also be elevated like HCV in this population. Therefore, the prevalence of HHV8 and HCV, as well as several potential infectious agents were analyzed in parallel in this population.

## 2. Materials and Methods

### 2.1. Study Setting and Populations

This cross-sectional study was conducted in Putuo district, Shanghai, China, from 2012 through 2013. All individuals, ≥18 years of age who had a history of drug use in the past six months and resided in Shanghai, were eligible for enrollment. Subjects were recruited consecutively using convenience sampling from a drop-in center (DIC) serving drug users in Shanghai. Potential subjects were not required to disclose their names for participation. They received modest monetary compensation for time spent for the interview.

The purpose and methods of the study were explained to all the participants. Informed consent procedures were carried out individually, and written consent was obtained from all the participants before any procedures were performed. This study was approved by the Institutional Review Board of Fudan University, China.

### 2.2. Data Collection

After obtaining written consent, participants were interviewed by a trained health professional using a standard survey questionnaire covering demographics, drug use practices and sexual risk behaviors. Interviews were administered in-person in a private location (such as methadone clinics). Completed questionnaires were placed in a large black bag containing other completed questionnaires to reassure the participants about confidentiality of the provided information.

### 2.3. Sample Collection

Venous blood was collected by experienced nurses using sterilized needles, syringes and tubes, and transferred to the laboratory within 2 h after collection. Plasma samples were stored at −80 °C until serological testing. All specimens were coded by a unique identification number given to each study participant and were analyzed by two experienced technicians without any knowledge of the study participants identities.

### 2.4. Specimen Testing

HIV serology. All plasma samples were screened for HIV antibody using an enzyme-linked immunosorbent assay (ELISA; Vironostika HIV Uni-Form II plus O ELISA Kit, Biomerieux Shanghai Company Ltd., Shanghai, China), according to the manufacturer’s instructions. All positive samples were further confirmed by Western blot assay (Genelabs Diagnostic, Singapore, Singapore).

HHV8 serology. Plasma samples were tested by immunofluoresence assay, as reported previously [19]. Briefly, two HHV8 serology tests were performed: first, BC-3 cells (HHV8 positive and Epstein-Barr virus negative B cell line, American Type Culture Collection, Manassas, VA, USA), stimulated by tetradecanoyl phorbol acetate (TPA) were fixed and permeabilized and used for monoclonal enhanced immunefluorescence assay. Second, *Spodoptera frugiperda* clone 9 expressing viral recombinant proteins, ORF73, ORF65, and ORF-K8.1, was used for testing. The procedure was similar to the BC-3 immunofluoresence assay. A sample was considered HHV8 seropositive only if it was positive at a standard serum dilution of 1:40 with both the BC-3 and *S. frugiperda* assay. Each slide was read independently by two experienced laboratory workers.

HBV and HCV serology. HBsAg was tested using an ELISA kit (Wantai Biotech Pharmacy Enterprise Co. Beijing, China). The test was performed following the procedures recommended by the manufacturer. Anti-HCV immunoglobulin G (IgG) antibody was tested to determine HCV infection status according to the manufacturer’s protocol (Wantai Biomedical, Beijing, China). All the plasma samples were blindly assayed in duplicate.

Syphilis Serology. Syphilis was screened by using a rapid plasma reagent test (Span Diagnostics Ltd., India), and confirmed by the *Treponema pallidum* hemaglutination test (TPHA, Syphagen TPHA, Biokit, Barcelona, Spain).

All the above serological tests were performed by the same two experienced technicians, with duplicate negative, positive, and blank controls being tested in parallel.

### 2.5. Statistical Analysis

Original questionnaires and laboratory testing results were entered and managed in EpiData3.0, and then transferred to a SAS database for further analyses. Demographic characteristics and risk behaviors were analyzed using descriptive statistics, *i.e.*, mean, median, and interquartile range (IQR) for continuous variables, and proportions for categorical variables.

HHV8 seroprevalence was computed using the normal approximation to a binomial distribution, and tabulated by sociodemographic characteristics of study subjects, followed by Pearson’s chi-squared tests to determine statistical significance. Initially, a univariate logistic regression analysis was conducted, followed by multivariate logistic regression analysis to explore associations between sexual behaviors and HHV8 seropositivity. Odds ratio (OR) and 95% confidence interval (95% CI) were used to determine whether a variable was associated with HHV8 infection. The nonparametric Mann-Whitney *U* test was used to assess the difference in the geometric mean titers (GMTs) of anti-HHV8 IgG between the HHV8 mono-infection and co-infection groups. A *p*-value less than or equal to 0.05 was considered to be statistically significant for all analyses. All statistical analyses were carried out using the SAS System for Windows (Cary, NC, USA), version 8.0.

## 3. Results

### 3.1. Socio-Demographic Characteristics of Participants

A total of 441 drug users were interviewed for this study, including 334 males and 107 females. The participants’ characteristics are summarized in Table 1. Most participants (99.5%) resided in the study area, and 97.1% were of the Han ethnicity. The age of the study population ranged from 20 to 61 years. Male participants were significantly older than the female participants (46.05 ± 8.31 *vs.* 43.31 ± 8.35, *p* = 0.003). Approximately, 95.7% participants had an education level above high school. Female participants were more likely to have a steady sex partner compared to the male participants. There were no significant sociodemographic differences between male and female in terms of residency, ethnicity and education level.

**Table 1:** Sociodemographic characteristics of study participants.

	Male (*n* = 334) No. (%)	Female (*n* = 107) No. (%)	Total (*n* = 441) No. (%)
**Residency (*p* = 1.000)**			
Local	332 (99.4)	107 (100.0)	439 (99.5)
Non-local	2 (0.6)	0 (0.0)	2 (0.5)
**Ethnicity (*p* = 0.526)**			
Han	325 (97.3)	103 (96.3)	428 (97.1)
Minority	9 (2.7)	4 (3.7)	13 (2.9)
**Age (years) (*p* = 0.005)**			
≤40	80 (24.0)	39 (36.4)	119 (27.0)
41–50	138 (41.3)	47 (43.9)	185 (42.0)
≥51	116 (34.7)	21 (19.6)	137 (31.1)
**Education (*p* = 0.107)**			
Primary or lower	18 (5.4)	1 (0.9)	19 (4.3)
Junior high	199 (59.6)	71 (66.4)	270 (61.2)
Senior high or college	117 (35.0)	35 (32.7)	152 (34.5)
**Steady partner (*p* = 0.012)**			
No	187 (56.0)	45 (42.1)	232 (52.6)
Yes	147 (44.0)	62 (57.9)	209 (47.4)
**HHV8-Ab (*p* = 0.842)**			
No	275 (82.3)	89 (83.2)	364 (82.5)
Yes	59 (17.7)	18 (16.8)	77 (17.5)
**HCV-Ab (*p* = 0.689)**			
No	127 (38.0)	43 (40.2)	170 (38.5)
Yes	207 (62.0)	64 (59.8)	271 (61.5)
**HIV-Ab (*p* = 0.427)**			
No	333 (99.7)	106 (99.1)	439 (99.5)
Yes	1 (0.3)	1 (0.9)	2 (0.5)
**Syphilis (*p* = 0.174)**			
No	327 (97.9)	102 (95.3)	429 (97.3)
Yes	7 (2.1)	5 (4.7)	12 (2.7)

The majority (67.1%) of the participants had a history of injection drug use, and used mainly heroin and/or cocaine. Among them, 3.7% reported ever sharing syringes. Meanwhile, about 7.3% of participants reported commercial sex behaviors, including four female respondents. In this study, 59.4% participants reported never using condom in commercial sex contact.

### 3.2. Seroprevalence of HIV, HHV8, HCV, and Syphilis

Of all the 441 participants, 77 (17.5%) were HHV8 seropositive. The majority (61.5%) of the study participants enrolled were HCV positive, while the HIV prevalence was extremely low with only two cases being HIV positive. Given this low frequency of HIV, it was not considered for further analysis. As shown in Table 2, among the 77 HHV8 positive individuals, 44 (57.1%) were coinfected with HCV, and one case coinfected with HCV and syphilis concurrently. With the 364 HHV8 negative individuals, 214 (58.8%) were infected only with HCV, 10 (2.8%) were dually infected with HCV and syphilis, and two were dually with HCV and HIV.

**Table 2:** Summary of coinfections by human herpesvirus 8 (HHV8), hepatitis C virus (HCV), Human immunodeficiency virus (HIV) and syphilis among study participants.

Co-infections	HHV8-Uninfected (N_1_ = 364)		HHV8-Infected (N_2_ = 77)	
	No.	Prevalence (%)	No.	Prevalence (%)
**None**	137	37.6	32	41.6
**Single pathogen**				
HIV	0	0.0	0	0.0
Syphilis	1	0.3	0	0.0
HCV	214	58.8	44	57.1
**Dual pathogens**				
HIV + HCV	2	0.5	0	0.0
HIV + Syphilis	0	0.0	0	0.0
HCV + Syphilis	10	2.8	1	1.3
**Total**	364	100.0	77	100.0

### 3.3. Correlates of HHV8 and HCV Seropositivity

With regard to HHV8 infection, the univariate analysis showed that few variables were associated with HHV8 positive status among study participants. No significant association was detected between HHV8 infection and any sociodemographic characteristics, drug use or sex behaviors. Moreover, no statistically significant association between HHV8 infection and HCV or syphilis were found either (data not shown). The lack of association between HHV8 seropositivity and potential variables remained in both male and female subgroups, when separated analyses were performed. No significant association was observed between HHV8 seropositivity and history of commercial sex, after adjusting for sociodemographic characteristics by using multiple logistic regression analysis. Although those who had commercial sex contact were more likely to be HHV8 positive (OR, 6.05; 95% CI 0.80–45.67), the association did not achieve significance (Table 3).

With regard to HCV infection, the univariate analysis indicated that education level, ever injected drugs, ever had commercial sex, and never use condom for commercial sex were associated with HCV infection. Moreover those who ever shared syringes were all found to be positive for HCV. Multivariate analysis, adjusting for potential confounder, indicated ever injected drug history were independently associated with HHV8 infection among participants (OR, 2.18, 95% CI 1.41–3.37). Meanwhile syphilis infection seemed to be associated with HHV8 seropositivity, but this association was not significant (OR 7.56, 95% CI 0.94–60.57). No additional risk factors were identified when men and women were analyzed as an independent group.

**Table 3:** Correlates of HHV8 seropositivity among study participants.

Risk Factors	No. HHV8 Infection/No. Tested (%)	aORs (95%CI) *	*p*-values
Ever injected drugs			
No	21/145 (14.5)	1.00	
Yes	56/296 (18.9)	1.50 (0.83–2.69)	0.181
Ever sharing syringe			
No	75/430 (17.4)	1.00	
Yes	2/11 (18.2)	1.06 (0.20–5.46)	0.949
Steady partner			
No	41/232 (17.7)	1.00	
Yes	36/209 (17.2)	0.97 (0.55–1.72)	0.922
Ever had sex in the past month			
No	53/288 (18.4)	1.00	
Yes	24/153 (15.7)	0.85 (0.42–1.71)	0.650
Condom use in the last sex intercourse			
Never or no sex	69/380 (18.2)	1.00	
Yes	8/61 (13.1)	0.57 (0.21–1.56)	0.273
Ever had commercial sex			
No	69/409 (16.9)	1.00	
Yes	8/32 (25.0)	6.05 (0.80–45.67)	0.081
Frequency of condom use in commercial sex			
Always or no sex	71/414 (17.1)	1.00	
Sometimes	3/8 (37.5)	1.59 (0.16–6.12)	0.120
Never	3/19 (15.8)	0.72 (0.06–8.32)	0.790
HIV			
No	77/439 (17.5)	-	
Yes	0/2 (0.0)	-	-
HCV			
No	32/170 (18.8)	1.00	
Yes	45/271 (16.6)	0.82 (0.48–1.41)	0.476
Syphilis			
No	76/429 (17.7)	1.00	
Yes	1/12 (8.3)	0.50 (0.06–4.09)	0.519

* aORs: adjusted Odds ratios and 95% CI: 95% confidence interval.

### 3.4. HHV8 Antibody Titers by Different Characteristics

**Figure 1:**
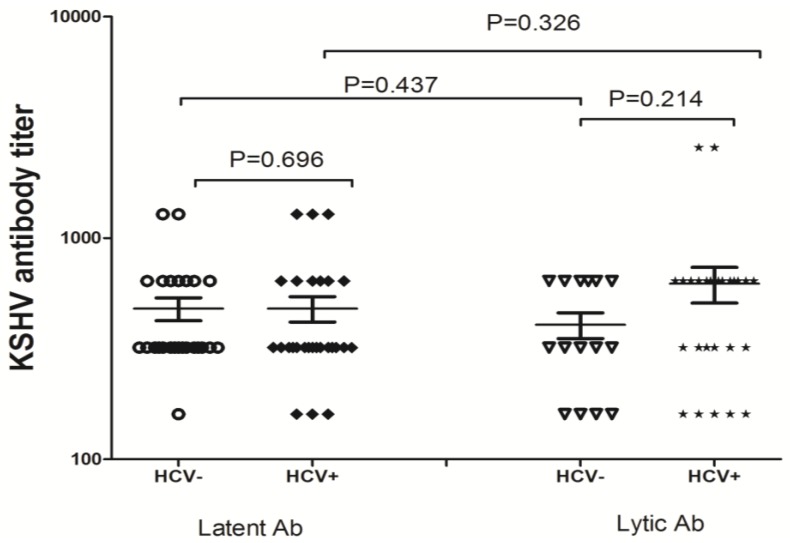
Anti-HHV8 IgG antibody titer among patients with HHV8 monoinfection *versus* patients with HHV8/HCV confection.

Since HHV8 and HCV coinfection is common in the study population, geometric mean titers (GMT) of antibodies to lytic and latent antigens of HHV8 were further compared according to their coinfection status. Figure 1 presents the distribution of HHV8 lytic and latent antibodies. The GMTs for HHV8 latent antibody were 480 (95% CI 362.5–597.5) and 480 (95% CI 351.0–609.0) in participants infected with HHV8 only and HHV8/HCV coinfection, respectively. While the GMTs for HHV8 lytic antibody were 405.3 (95% CI 290.0–520.7) and 622.2 (95% CI 387.8–856.6) for HHV8 mono infection and HHV8/HCV coinfection, respectively. Overall, the pairwise comparison indicated that neither lytic nor latent antibodies significantly differed across the two groups (for lytic antibody, Mann-Whitney *U* = 158.0, *p* = 0.214; for latent antibody Mann-Whitney *U* = 318.0, *p* = 0.695). Similarly, no significant differences for either lytic or latent antibody were observed with each group (for HHV8 group, Mann-Whitney *U* = 161.5, *p* = 0.437; for HHV8/HCV group Mann-Whitney U = 310.5, *p* = 0.326) (Figure 1).

## 4. Discussion

The present study aimed to address the status of HHV8 infection amongst a group of drug users from mainland China. In the present study, a moderate seroprevalence (17.5%) of HHV8 was identified, which is relatively lower than that (32.7%) among men who have sex with men (MSM), but higher than the general population in China [20]. Meanwhile, their HCV infection status was evaluated in parallel, and as expected a high HCV seroprevalence (61.5%) was observed among the study participants. This high seroprevalence of HCV among drug users is in accordance with findings from previous studies in China [15,16].

Of importance, the results of this study suggest that HHV8 seropositivity was not directly associated with injection drug use behaviors, or with HCV and syphilis infections in the participants. This is consistent with two previous reports one among a cohort of Netherlands drug users, and the other from a minority population in China [14,21]. Interestingly, although sexual behaviors were found to be associated with HHV8 seropositivity [18,22,23], the association was not well established in the present study. Specifically, there was no significant association between commercial sex and HHV8 seropositivity. Furthermore the seropositivity of HHV8 was not associated with syphilis, a typical sexually transmitted disease either, in the present study. This discrepancy could possibly be explained by the fact that the majority of the participants were heterosexual individual, and the possibility of HHV8 transmission via heterosexual contacts is still controversial. In this regard, more extensive studies are needed to address the association of sexual contact and HHV8 transmission in this population.

As expected, a high HCV prevalence was detected and injection drug use behaviors were found to be independent risk factors for HCV seropositivity. Most participants who have injected drugs and shared needles were seropositive for HCV, demonstrating that the blood-to-blood contact is highly efficient in transmitting HCV. Therefore, if the needle sharing and blood-to-blood contact is a major risk for HHV8 transmission, the observed prevalence of HHV8 should have been higher, and similar epidemiologic profiles should have been observed for HHV8 and HCV. The obvious difference in the prevalence of HHV8 and HCV infection in the study population, however, suggested that these two viruses should use distinct routes for efficient viral transmission. Interestingly, only a small proportion of the study participants self-reported needle sharing experience, possibly due to the effectiveness of the needle exchange program in reducing blood borne infectious disease transmission in China. Previous studies have also shown that indirect drug sharing and drug preparation practices, such as splitting drugs prepared by a user with subsequent transfer of the prepared drug from one syringe to a second syringe for another user, sharing cotton, filters, cooker, water, and water containers, are associated with HCV transmission [24,25,26,27]. These observations suggest that HHV8 is not transmitted efficiently through needle sharing or other behaviors among drug users. Therefore, it is likely that HCV, but not HHV8, could be efficiently transmitted through contaminated utensils during drug use practices. Taken together, findings from the present study, along with our previous study conducted in a community with high risks of blood borne infections particularly HCV, demonstrate that HHV8 and HCV do not share the same transmission routes [28]. This divergent characteristic between HHV8 and HCV further confirms that blood borne transmission is not a predominant transmission route for HHV8 but the possibility of blood borne transmission could not be completely ruled out.

This study is also subjected to certain limitations. First, the subjects in this study were recruited using a convenience sampling approach and the findings may not be representative of the whole drug-using population in China. Second, like most studies on sexual and drug use behavior, this study is potentially subjected to socially desirable responding or reporting bias. Third, only asymptomatically infected HHV8 individuals were recruited. Therefore, it is difficult to delineate the potential relationships between host lytic and latent antibody response to HHV8 antigens with KS risk. Nonetheless, in the present study, the parallel analysis of HHV8 and HCV serving as control for blood borne transmission, our findings could provide important information to a better understanding of the HHV8 epidemiology in China.

In conclusion, HHV8 seroprevalence is relatively low in the drug user and mirrors the low KS disease burden in this population in China. Injection drug use and needle sharing practice were not found to be a risk factor for HHV8 transmission among the drug users in the current study. In contrast, HCV was highly prevalent among this population and confirms that injection drug use behaviors are independent risk factors of HCV infection. Given the importance of this population in HIV prevention, more extensive study regarding HHV8 transmission and the effects of infection in this risk group is warranted.
